# Identification and experimental validation of mitochondria-related genes biomarkers associated with immune infiltration for sepsis

**DOI:** 10.3389/fimmu.2023.1184126

**Published:** 2023-05-09

**Authors:** Qi Shu, Han She, Xi Chen, Like Zhong, Junfeng Zhu, Luo Fang

**Affiliations:** ^1^ Zhejiang Cancer Hospital, Institute of Basic Medicine and Cancer (IBMC), Chinese Academy of Sciences, Hangzhou, China; ^2^ Department of Anesthesiology, Daping Hospital, Army Medical University, Chongqing, China; ^3^ Department of Gastroenterology, The Affiliated Hospital of Qingdao University, Qingdao, China

**Keywords:** mitochondria, sepsis, machine learning algorithm, immune cell infiltration, mito-chondrial quality imbalance

## Abstract

**Background:**

Sepsis remains a complex condition with incomplete understanding of its pathogenesis. Further research is needed to identify prognostic factors, risk stratification tools, and effective diagnostic and therapeutic targets.

**Methods:**

Three GEO datasets (GSE54514, GSE65682, and GSE95233) were used to explore the potential role of mitochondria-related genes (MiRGs) in sepsis. WGCNA and two machine learning algorithms (RF and LASSO) were used to identify the feature of MiRGs. Consensus clustering was subsequently carried out to determine the molecular subtypes for sepsis. CIBERSORT algorithm was conducted to assess the immune cell infiltration of samples. A nomogram was also established to evaluate the diagnostic ability of feature biomarkers via “rms” package.

**Results:**

Three different expressed MiRGs (DE-MiRGs) were identified as sepsis biomarkers. A significant difference in the immune microenvironment landscape was observed between healthy controls and sepsis patients. Among the DE-MiRGs, *NDUFB3* was selected to be a potential therapeutic target and its significant elevated expression level was confirmed in sepsis using *in vitro* experiments and confocal microscopy, indicating its significant contribution to the mitochondrial quality imbalance in the LPS-simulated sepsis model.

**Conclusion:**

By digging the role of these pivotal genes in immune cell infiltration, we gained a better understanding of the molecular immune mechanism in sepsis and identified potential intervention and treatment strategies.

## Introduction

Sepsis is a fatal syndrome resulting from multiple organ failure caused by an inappropriate host response to infection. Despite a significant decline in sepsis mortality due to improvements in life support techniques, long-term ICU stays remain on the rise, resulting in a significant socioeconomic burden (1, 2). Despite significant advances in diagnosis and treatment, the incidence of sepsis continues to increase due to an incomplete understanding of the pathogenesis due to individual heterogeneity and the complexity of the infection (3). At the same time, there are few studies on the factors related to long-term mortality in patients with sepsis, and risk stratification is unclear (4, 5). Therefore, there is a need to further search for prognostic factors, more accurate risk stratification for sepsis, and more sensitive and specific diagnostic and therapeutic targets.

In recent years, the role of mitochondria beyond energy supply has received increasing attention. Danger signals actively secreted or passively released by dead or damaged cells are known as damage-related molecular patterns (DAMPs). DAMPs activate the immune system by activating classical pattern recognition receptors (PRRS)or non-PRR pathways, including ion channels and G-protein-coupled receptors (6). ATP, the main form of energy produced by mitochondria, is a DAMP. Damage-induced release of mitochondria and their contents can increase local ATP levels, thereby enhancing the killing effect of macrophages in sepsis through P2X7 and P2X4 receptors (7, 8). In addition to ATP, various components of mitochondria can be used as DAMPs, including transfactor A, cytochrome c, succinate, mitochondrial (TFAM), cardiolipin, and mtRNA (9). These mitochondrial damage-associated molecular patterns (mtDAMPs) can be stimulated by lipopolysaccharide secretion by monocytes in extracellular vesicles (10). mtDAMP released into circulation is recognized by the immune system and drives an inflammatory response (11). Additonally, during sepsis process, mitochondrial stress leads to mitochondrial membrane potential decreases, resulting in impaired membrane integrity (12). This mitochondrial damage causes mitochondrial DNA (mtDNA) leaking into the cytoplasm and acting as a key DAMP. By affecting the respiratory chain, enhancing oxidative stress and inflammatory response, inducing cell apoptosis, mitochondrial damage leads to cell dysfunction and tissue damage, and further aggravates mitochondrial dysfunction, thus forming a feedback loop (13).

Uncontrolled inflammation following severe trauma is often one of the key factors leading to organ damage and poor prognosis. An increasing number of reports have hinted at the important roles of mtDNA and mtDAMP in sepsis (14). mtDAMPs can induce a strong inflammatory response and septicaemia - like symptoms. mtDAMPs are present in trauma patients. Animals treated with mtDAMP showed an exaggerated inflammatory response triggered by mtDAMP (14). Traumatized patients had higher plasma mtDNA levels (15, 16) than healthy subjects. Moreover, mtDNA levels were closely associated with post-injury complications (17). Plasma levels of circulating mtDNA were significantly higher in patients with severe sepsis/septic shock than in patients with postoperative inflammation or trauma (18, 19). Thus, the plasma concentration of mtDNA was considered to be an independent predictor of post-traumatic SIRS (20). mtDNA may be better than lactate concentration or even SOFA score in predicting mortality after admission in sepsis patients (21). Further studies of both mtDNA and mtDAMPs in sepsis will greatly improve our understanding of sepsis pathogenesis.

In this study, we aim to use machine learning algorithms to identify new mitochondria-related genes (MiRGs) in sepsis. Three MiRGs (*BCKDHB*, *LETMD1*, and *NDUFB3*) were screened out. More information about the role of MiRGs in immune infiltration was further explored. After the verification of high expression of *NDUFB3* with our sepsis clinical specimens, the effect of *NDUFB3* on mitochondria was tested with a confocal microscope. The expression of *NDUFB3* was inhibited by small interfering RNA technology and the mitochondrial function was significantly reduced in the sepsis model. This study provides new ideas and targets for the intervention and treatment of sepsis.

## Materials and methods

### Sepsis datasets collection

Three public gene expression matrices (GSE54514, GSE65682, and GSE95233), comprising of gene expression data from sepsis patients (SP) and healthy controls (HC), were obtained from the Gene Expression Omnibus (GEO) databases. The GSE65682 dataset (GPL13667, [HG-U219] Affymetrix Human Genome U219 Array), consisting of 42 healthy samples and 760 sepsis samples, served as the training cohort, while the GSE54514 (GPL6947, Illumina HumanHT-12 V3.0 expression beadchip) and GSE95233 (GPL570, [HG-U133_Plus_2] Affymetrix Human Genome U133 Plus 2.0 Array) datasets, which included 58 healthy samples and 229 sepsis samples, were utilized as the test cohort. The R script “sva” was employed to normalize the data and eliminate any batch effects present in the three datasets (20). The definition of differentially expressed genes (DEGs) was established at |Fold change| ≥ 2, p (p. adjust) < 0.05. Mitochondria-related genes (MiRGs) were collected from the Mito-Carta, MitoMiner, IMPI 2, and UniProt databases.

### WGCNA and machine learning algorithm

Weighted gene co-expression network analysis (WGCNA) was employed in this study to identify the pivotal gene module associated with sepsis and healthy controls. Initially, all samples underwent clustering to exclude anomalous samples. Next, with the application of the scale-free topology model fit (R^2 = ^0.85), a network was constructed with a soft threshold (power) of 9 (β). The genes were subsequently separated into distinct modules and clustered in a tree, which were then merged into the final module. Pearson correlation algorithm was employed to calculate the correlation between each gene module. Eventually, the association between clinical features and gene modules was estimated, and the most relevant modules for the following analysis were selected. The least absolute shrinkage and selection operator (LASSO) logistic regression and random forests (RF) were employed for feature selection to screen diagnostic markers for sepsis. The LASSO algorithm was conducted using the “glmnet” package, while the RF algorithm was implemented as tree-based methods for classification and regression analysis. In this study, variables with the minimum log lambda of LASSO were considered as characteristics variables, and the importance threshold for selecting crucial variables using RF was set at 3. The common genes from LASSO and RF were obtained by Venn plot and used for further analysis.

### Exploration of functional enrichment in (DE-MiRGs)

We utilized the “clusterProfiler” and “ggplot2” packages to perform enrichment analyses of DE-MiRGs using Gene Ontology (GO) and the Kyoto Encyclopedia of Genes and Genomes (KEGG) (22). Furthermore, we applied GSEA to enrich the DEGs into distinct functional signaling pathways for HC and SP groups.

### Nomogram development based on the diagnostic biomarkers

A nomogram model, which is based on the differentially expressed miRNA-regulated genes (DE-MiRGs), was constructed using the R package “rms”, to estimate the diagnostic probability of sepsis patients. To validate the diagnostic capacity of the nomogram, a receiver operating characteristic (ROC) curve was plotted. The nomogram scores were computed using the following parameters: -2.548 x BCKDHB + -5.454 x LETMD1 + 4.507 x NDUFB3.

### Immune infiltration and consensus clustering analysis

Based on the expression matrix of each samples in the training cohort (GSE65682), the CIBERSORT algorithm was employed to evaluate the immune infiltration. Following the computation of marker genes for 22 immune cells, the relative proportion of the 22 immune cells was obtained. To investigate the correlation between infiltrating immune cells and 3 diagnostic biomarkers, Pearson’s correlation was conducted using the “ggplot2” R package. The 3 diagnostic DE-MiRGs were utilized for consensus clustering, with a maximum K of 9, via the R package “ConsensusClusterPlus”. Based on the optimal classification of K = 2, sepsis patients were classified into 2 molecular subtypes for further analysis. “ggplot2” script was utilized to exhibit the distribution pattern of HC and SP groups in a PCA plot based on the 22 immune cells proportion.

### Clinical samples

The clinical blood samples used in this study were obtained from 30 sepsis patients and 15 healthy volunteers at Daping Hospital (Chongqing, China) (**Supplementary Table 1**). The study was approved by the Ethics Committee of the Army Medical University and was registered with the Chinese Clinical Trial Registry (ChiCTR2200055772). All procedures were carried out under the approval of the Ethics Committee, and informed consent was obtained from all patients prior to participation.

### Reagents and cell culture and treatment

The Mito-tracker was procured from Thermo Fisher Scientific (Waltham, MA, USA). The ROS assay kit, ATP detection kit, and JC-1 enhanced mitochondrial membrane potential assay kit were purchased from Beyotime (Shanghai, CHINA). The siRNA for NDUFB3 was generated by Obio Technology (Shanghai, CHINA), and the target sequence of siNDUFB3 was 5′- GAUUAUAGACAAUGGAAGATT -3′. H9C2 cells were obtained from the American Type Culture Collection (ATCC), located in Manassas, VA, USA, and cultured in DMEM supplemented with 10% FBS and 1% antibiotics in a 5% CO2/95% air atmosphere, at 37°C (23). To construct an *in vitro* sepsis model, H9C2 cells were stimulated with 1μg/ml LPS, purchased from Sigma (St. Louis, MO, USA), for 12 hours.

### Immunofluorescence

The cells were introduced into the confocal chamber and incubated with Mito-tracker (diluted to a ratio of 1:10,000), DCFH-DA (diluted to a ratio of 1:1,000), and JC-1 (diluted to a ratio of 1:1,000) at a temperature of 37°C for a period of 30 minutes. Following this, observations were made of the mitochondrial morphology, reactive oxygen species (ROS), and mitochondrial membrane potential utilizing a laser confocal microscope (Leica SP5, Germany). The mitochondrial length was subsequently analyzed utilizing a mitochondrial network analysis (MiNA) toolset, which was included in the ImageJ software (https://fiji.sc/).

### qRT-PCR analysis

The blood RNA was extracted using the PureLink™ blood total RNA extraction kit (Invitrogen).Then, the extracted RNA was then reverse transcribed into cDNA libraries using the Bestar™ qPCR RT Kit (DBI Bioscience), and fluorescent quantitative PCR reactions were performed using the Bestar^®^ SYBRGreen qPCR master mix (DBI Bioscience). Actin was handled as an internal reference. Primers used in these experiments were listed in **Supplementary Table 2**.

### Statistical analysis

Statistical analyses were performed using R (version 4.1.1), GraphPad Prism (version 8.0.1), and SPSS 17.0 (SPSS Inc., Chicago, IL, USA). Cell study data were repeated in a minimum of three independent experiments. In this study, statistical differences between the two groups were tested using the T test and Wilcoxon rank-sum test. One-way ANOVA analysis was used between multiple groups. All data are presented as the mean ± standard deviation (SD) and statistical significance was considered at p < 0.05.

## Results

### DEGs screening and GSEA analysis

**Figure 1:**
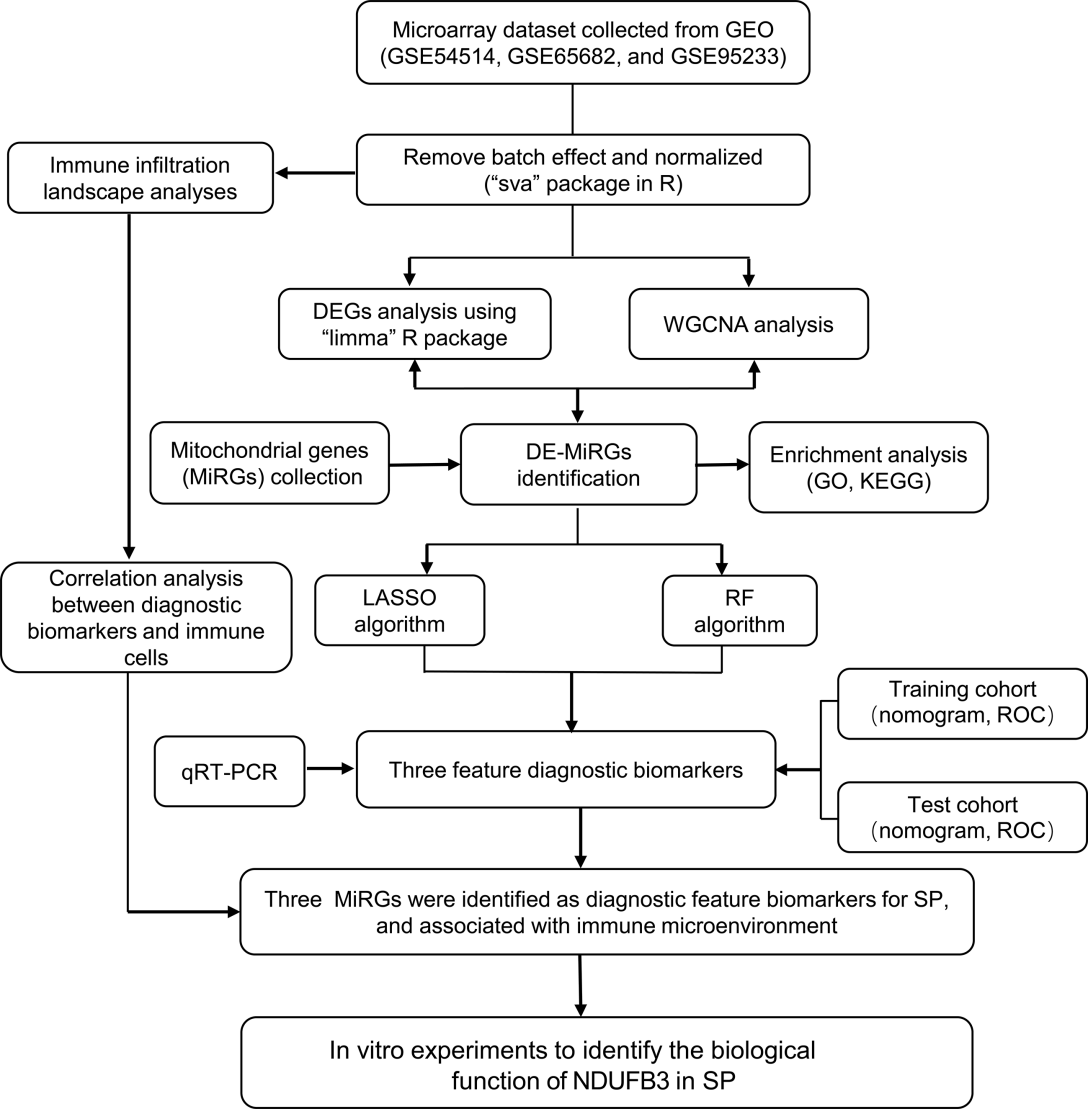
Diagram of the Study flow.

**Figure 2:**
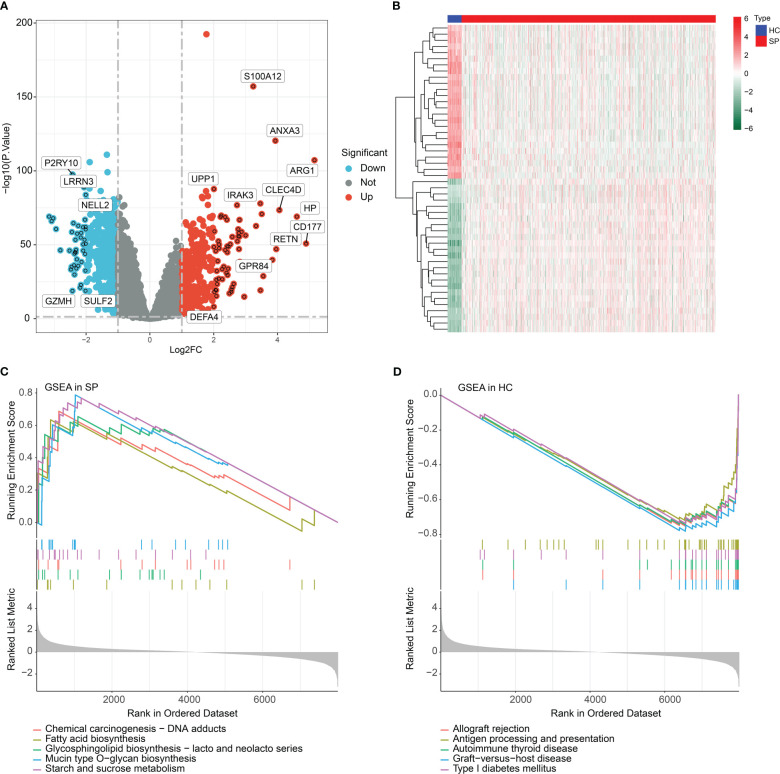
Identification of DEGs and GSEA functional enrichment analysis. **(A)** Volcano plot of DEGs in HC and SP groups. The threshold of screening DEGs is set at |fold change| ≥ 2 and p (p.adjust) < 0.05. Turquoise dots represent down-regulated genes and red dots represents up-regulated genes. **(B)** Analysis of top 25 up- and down-regulated genes in HC and SP group. **(C, D)** GSEA analysis of DEGs in HC and SP group.

Three datasets were collected from the GEO database (GSE95233, GSE65682, GSE54514) in this study. After removing the batch effect and data normalization, we collected 100 healthy samples (HC) and 989 sepsis samples (SP) for the subsequent analyses (**Figure 1**). In the training cohort (GSE65682), under the screening condition set at |fold change| ≥ 2 and p-value (p.adjust) < 0.05, 744 DEGs were obtained, including 398 down- and 346 up-regulated DEGs (**Figure 2A**). The heatmap diagram showed the expression of the top 25 up- and down-regulated DEGs in HC and SP groups (**Figure 2B**). The analysis of GSEA suggested that the DEGs in the SP group were greatly enriched in chemical carcinogenesis-DNA adducts, fatty acid biosynthesis, glycosphingolipid biosynthesis-lacto and neolacto series, mucin type O−glycan biosynthesis, and starch and sucrose metabolism; however, we found that the DEGs in HC group were remarkably enriched in immune-related signaling pathways, including antigen processing and presentation and allograft rejection (**Figures 2C, D**).

### Construction of WGCNA

**Figure 3:**
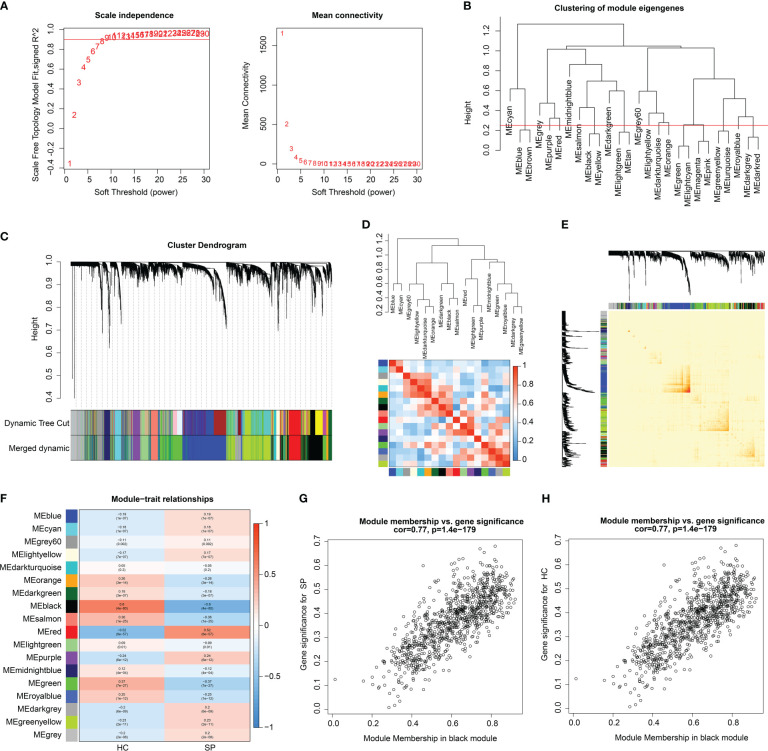
WGCNA analysis to select characteristics gene module for SP. **(A)** Scale free topology model fit (R^2 = ^0.85) and mean connectivity. **(B)** Clustering of module genes. **(C)** Cluster dendrogram for selecting gene modules. **(D)** Association between the gene modules. **(E)** Correlation analysis of transcriptome in different modules. **(F)** Heatmap analysis of 18 modules and clinical features (HC, SP). **(G, H)** Module membership *vs*. gene significance for SP and HC in black module.

The training cohort (GSE65682) was utilized to develop a WGCNA network. The 42 HC samples and 760 SP samples were clustered under a set threshold condition to exclude abnormal samples. Under the filter of scale-free topology (R^2^ > 0.85), the soft threshold (power) for scale independence was selected as β = 9 (**Figure 3A**). With the height of clustering of module eigengenes set at 0.25, a total of 18 gene modules were obtained for further analysis (**Figure 3B**). The cluster dendrogram indicated the height of each module which was cut by the dynamic tree and merged into modules (**Figure 3C**). The correlation heatmap suggested that there was no apparent correlation between each module (**Figure 3D**). In addition, transcriptional correlation analysis within each module showed that there was no significant association between modules, showing the reliability of module descriptions (**Figure 3E**). The relationship of gene modules and clinical features illustrated that module black was negatively correlated with SP (r = -0.6, p = 4e-80), and positively correlated with HC (r = 0.6, p = 4e-80); module red was negatively associated with HC (r = -0.52, p = 8e-57), and positively correlated with SP (r = 0.52, p = 8e-57); module green was positively correlated with HC (r = 0.37, p = 7e-27), and negatively correlated with SP (r = -0.37, p = 7e-27, **Figure 3F**). According to the correlation coefficient, module black was identified as the most characteristic module. The scatter plot indicated that the module membership versus gene significance showed a high correlation of HC and SP (r = 0.77, p = 1.4e-179), and the genes in this module were collected for further analysis (**Figures 3G, H**).

### Identification of DE-MiRGs and functional enrichment analysis of pivotal module genes

**Figure 4:**
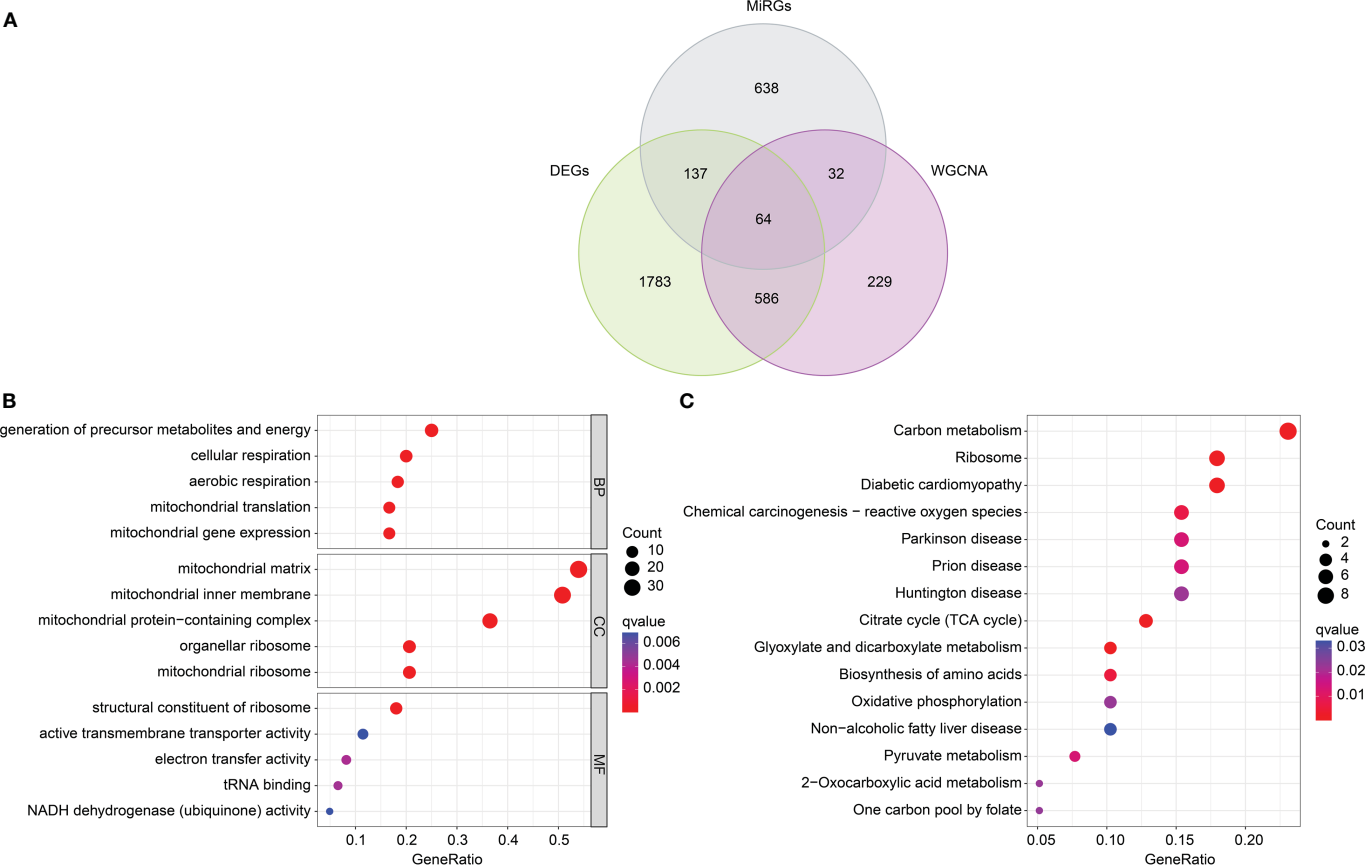
DE-MiRGs screening and function enrichment analysis. **(A)** Identification of pivotal DE-MiRGs in black module. **(B)** GO enrichment analysis of DE-MiRGs. **(C)** KEGG pathway analysis of DE-MiRGs.

Based on the different analysis and WGCNA (black module), 64 overlapping genes were identified as pivotal DE-MiRGs by the Venn diagram (**Figure 4A**) (24). We utilized function enrichment analysis to explore the potential molecular biological function of pivotal DE-MiRGs for SP. The analysis of GO enrichment illustrated that those pivotal DE-MiRGs were associated with the generation of precursor metabolites and energy, cellular respiration, mitochondrial matrix, and structural constituent of ribosome (**Figure 4B**). The KEGG analysis of pivotal DE-MiRGs was linked with carbon metabolism, ribosome, diabetic cardiomyopathy, and chemical carcinogenesis-reactive oxygen species (**Figure 4C**).

### Identification of feature biomarkers

**Figure 5:**
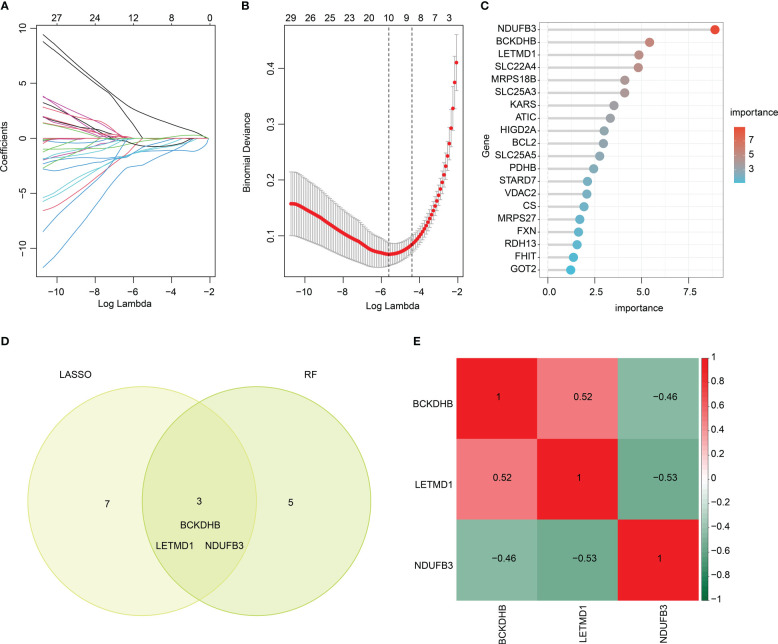
Feature biomarkers selection via machine language algorithms. **(A, B)** LASSO analysis to screen key DE-MiRGs. **(C)** RandomForest (RF) analysis of key DE-MiRGs, the filter condition for screening feature variates is set at: importance > 3. **(D)** Venn analysis of LASSO and RF. The overlapping genes are considered as feature biomarkers. **(E)** Correlation heatmap of *BCKDHB*, *LETMD1*, and *NDUFB3*. Green color represents negative correlation, red color represents positive correlation.

We performed two machine learning algorithms to select the feature DE-MiRGs for SP. The LASSO algorithm showed the minimum lambda of DE-MiRGs, and 10 characteristic variates were obtained (**Figures 5A, B**). Random forest (RF) algorithm result identified 8 feature DE-MiRGs for further analysis (**Figure 5C**). According to LASSO and RF algorithms, three overlapping genes were identified as feature biomarkers, including *BCKDHB*, *LETMD1*, and *NDUFB3* (**Figure 5D**). As displayed in **Figure 5E**, a remarkable association was observed between the three feature biomarkers; *BCKDHB* was positively associated with *LETMD1* and negatively associated with *NDUFB3*; *NDUFB3* was negatively correlated with *LETMD1*.

### Validation of feature biomarkers and effectiveness evaluation

**Figure 6:**
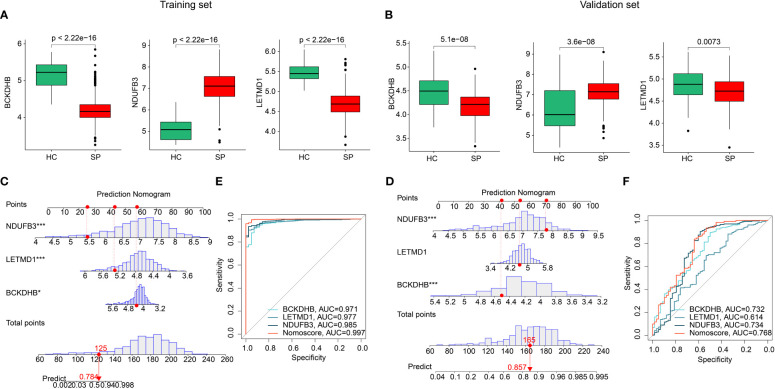
Validation of the expression of feature biomarkers and effectiveness evaluation. **(A)** The expression of *BCKDHB*, *LETMD1*, *NDUFB3* in training cohort (GSE65682). **(B)** Validation of *BCKDHB*, *LETMD1*, *NDUFB3* in validation cohort (GSE95233, GSE54514). **(C, D)** Nomogram construction and ROC curve of three gene signatures in GSE65682. **(E, F)** Nomogram construction and ROC curve of three gene signatures in GSE95233 and GSE54514.

Two separate cohorts were adopted to validate the expression and diagnostic effectiveness of feature biomarkers. In the training cohort (GSE65682) and validation cohort (GSE95233, GSE54514), the expression of three feature biomarkers suggested the HC group had higher expression of *BCKDHB*, *LETMD1*, and lower expression of *NDUFB3* (**Figures 6A, B**). Moreover, a nomogram model was established based on three gene signatures to evaluate the diagnostic effectiveness of SP in both cohorts. The results of the nomogram illustrated a satisfactory diagnostic ability of *BCKDHB*, *LETMD1*, *and NDUFB3* for SP (**Figures 6C, E**). The ROC analysis in the training cohort suggested that the AUC of three feature biomarkers (*BCKDHB*, *LETMD1*, *NDUFB3*) and nomogram score was 0.971, 0.977, 0.985, and 0.997, respectively (**Figure 6D**). The ROC analysis of feature biomarkers (*BCKDHB*, *LETMD1*, *NDUFB3*) and nomogram score in the validation cohort displayed that the AUC was 0.732, 0.614, 0.734, and 0.768 (**Figure 6F**). These results demonstrate a satisfactory diagnostic effectiveness of three feature biomarkers that could be used for clinical precision diagnosis of SP.

### Analysis of immune microenvironment landscape

**Figure 7:**
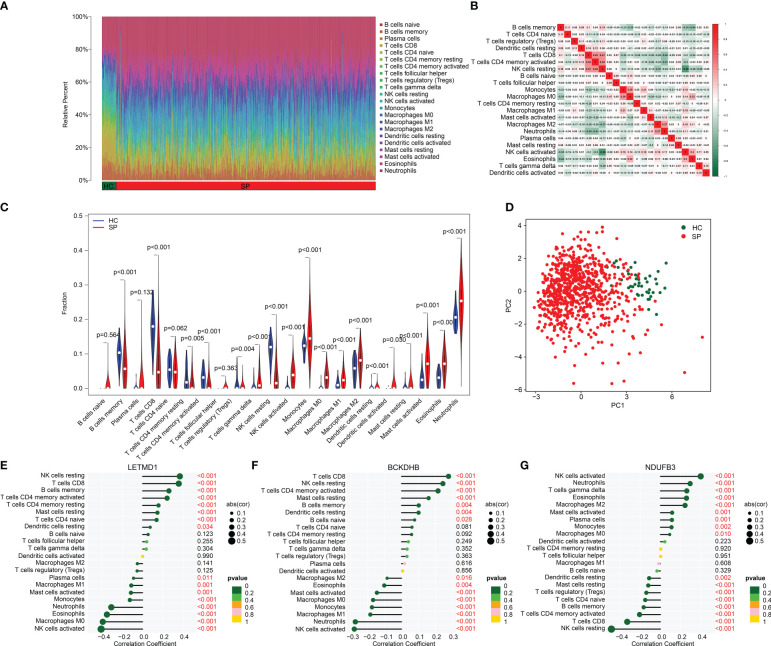
Analysis of immune microenvironment landscape in HC and SP groups. **(A)** Immune cells assessment between HC and SP groups. **(B)** Analysis of correlation in 22 type immune cells. **(C)** Violin diagram of 22 type immune cells in HC and SP groups. **(D)** PCA plot showed a different distribution pattern in HC and SP. **(E–G)** Correlation analysis of three diagnostic biomarkers (*BCKDHB*, *LETMD1*, and *NDUFB3*) and immune microenvironment.

By assessing the signature of 22 immune cell subtypes, we calculated the relative percent of 22 immune cells in the HC and SP groups based on the CIBERSORT algorithm (**Figure 7A**). Between the 22 immune cells, a significant correlation was observed in a heatmap; NK cells resting was negatively correlated with NK cells activated (r = -0.68), but positively correlated with T cells CD8 (r = 0.41); T cells CD4 memory activated was positively correlated with T cells CD8 (r = 0.42); B cells memory was negatively correlated with eosinophils (r = -0.44), NK cells activated (r = -0.35) and macrophages M0 (r = -0.35) (**Figure 7B**). Quantitative data revealed a great difference between HC and SP groups in most immune cells, such as B cells memory, T cells CD8, T cells CD4 memory resting, T cells CD4 memory activated, and T cells regulatory (Tregs) (**Figure 7C**). PCA plot illustrated a significant classification of immune cells in HC and SP groups (**Figure 7D**). Moreover, the correlation analysis of three feature biomarkers and the immune microenvironment landscape suggested that *BCKDHB*, *LETMD1*, and *NDUFB3* were greatly correlated with 22 immune cells (**Figures 7E–G**). Collectively, these findings demonstrate a significant difference between HC and SP groups in the immune microenvironment and closely associated with *BCKDHB*, *LETMD1*, and *NDUFB3*.

### Consensus clustering analysis of three diagnostic biomarkers for SP

**Figure 8:**
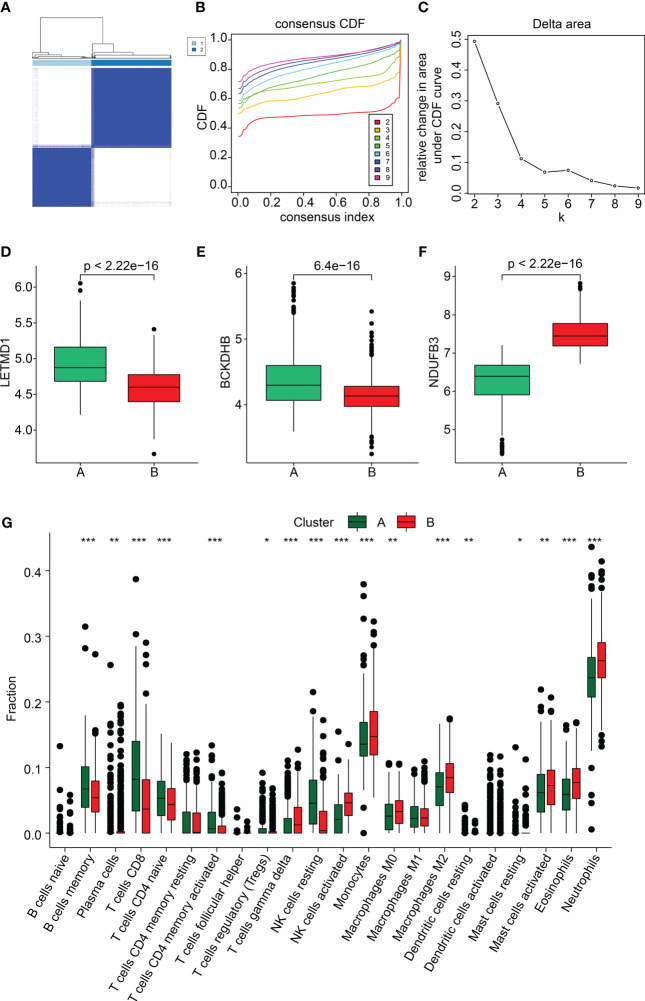
Subgroup analysis of SP samples based on three feature biomarkers. **(A–C)** Consensus clustering analysis. **(D–F)** The expression of *BCKDHB* and *LETMD1*, and *NDUFB3* in both cluster subgroups. **(G)** Immune microenvironment analysis of subgroups. *p<0.05, **p<0.01, ***p<0.001.

We performed a consensus clustering analysis to cluster the SP samples into different molecular subgroups. Based on the three diagnostic biomarkers, two optimal classifications were obtained for SP (**Figures 8A–C**). The quantitative data indicated that the samples in Cluster A had higher expression of *BCKDHB* and *LETMD1*, whereas the expression of *NDUFB3* was higher in Cluster B (**Figures 8D–F**). The immune cell assessment result illustrated that the immune microenvironment of samples in both cluster classifications was greatly different, such as B cells memory, T cells CD8, T cells CD4 naïve, and neutrophils (**Figure 8G**). The analysis of GSEA suggested that the DEGs in the Cluster A group were greatly enriched in Antigen processing and presentation and graft versus host disease, while the DEGs in Cluster B group were remarkably enriched in Glycosphingolopid biosynthesis and O-glycan biosynthesis (**Supplementary Figure 1**). These findings demonstrate that the SP samples could be accurately classified into different molecular subgroups based on the three feature biomarkers and notably correlated with the immune microenvironment.

### Effects of NDUFB3 on mitochondrial dysfunction in sepsis

**Figure 9:**
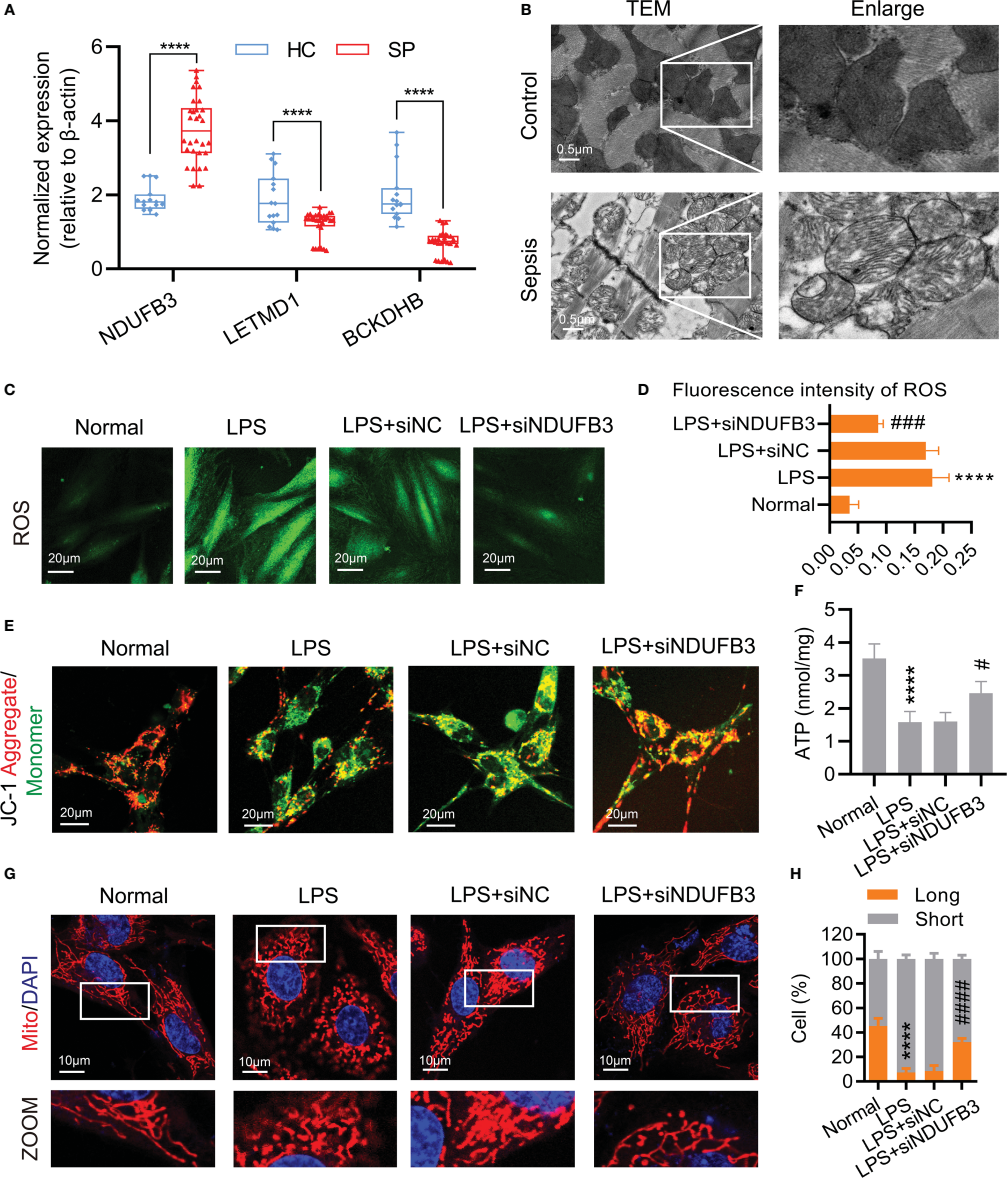
*NDUFB3* inhibition attenuated mitochondrial quality imbalance after sepsis. **(A)** qPCR was conducted to examine the expression of *NDUFB3*, *LETMD1* and *BCKDHB* in 30 SP and 15 HC samples. ****P< 0.0001 as compared with the SP group. **(B)** TEM images showed mitochondria cristae damage in heart tissues (bar = 0.5 μm) (n=3). **(C)** ROS staining immunofluorescence reflected the oxidative stress in H9C2 cells (bar = 20 μm) (n=3). **(D)** The fluorescence intensity of ROS. **(E)** JC-1 aggregate/monomer reflected the mitochondrial membrane potential in H9C2 cells (bar = 20 μm) (n=3). **(F)** The concentration of ATP (n=3). **(G)** Representative images of mitochondrial morphology in H9C2 cells (bar = 10 μm) (n=3). **(H)** Ratio (long/short) of mitochondria (long (> 8 µm) and short ≤ 8 µm) was quantified by ImageJ. ^****^ *P*< 0.01 as compared with the normal group, ^#^ *P*< 0.05 as compared with the LPS group, ^###^ *P*< 0.001 as compared with the LPS group, ^####^ *P*< 0.0001 as compared with the LPS group. all data are presented as the mean ± SD.

In order to further validate the expressions of 3 diagnostic biomarkers in sepsis, we collected the serum of 30 septic patients (SP) and 15 healthy controls (HC) for quantitative real-time PCR (qPCR). The results of qPCR showed that the expression of *NDUFB3* was higher in sepsis, whereas the expression of *LETMD1* and *BCKDHB* were lower in SP than in HC (**Figure 9A**). At the same time, the results of TEM showed that, as compared with the control group, the mitochondria ridge in sepsis was disordered, and broken with obvious vacuolar degeneration (**Figure 9B**).

We further explored the possible roles of DE-MiRGs dysregulation on mitochondrial function in sepsis. As the highest AUC among the three diagnostic biomarkers, we mainly focused on the role of *NDUFB3*. H9C2 cells were stimulated with LPS to mimic the *in vitro* sepsis model (25). The fluorescence intensity of ROS (**Figures 9C, D**) was significantly increased, indicating that oxidative stress occurred in the LPS group. The mitochondrial membrane potential and ATP reduced in H9C2 cells represented the dysfunction of mitochondria (**Figures 9E, F**). The mitochondrial morphology of H9C2 cells was significantly fragmented, confirming the existence of mitochondrial damage after sepsis (**Figures 9G, H**). Negative control (siNC) could not improve the mitochondrial function and morphology of H9C2 cells after being treated with LPS (**Figures 9C–H**). *NDUFB3* inhibition by siNDUFB3 (**Supplementary Figure 2**) could significantly attenuate mitochondrial dysfunctions of H9C2 cells, showing a decreased ROS level, increased mitochondrial membrane potential and ATP (**Figures 9C–F**). Besides, *NDUFB3* inhibition by siNDUFB3 could significantly improve mitochondrial morphology in LPS-treated H9C2 cells (**Figures 9G, H**). Overall, our results suggested that *NDUFB3* is highly expressed in sepsis and plays a vital role in the mitochondrial quality imbalance in LPS -treated H9C2 cells.

## Discussion

Sepsis is a potentially life-threatening condition caused by the spread of bacteria or toxins in the bloodstream. Mitochondria, the powerhouses of the cell, play a crucial role in the immune response to sepsis by releasing signals that initiate an inflammatory response and energy production to fight the infection (9). However, mitochondrial dysfunction can exacerbate the severity of septicemia and increase the risk of mortality. In this study, we found the mitochondria ridge in sepsis patients was disordered, broken with obvious vacuolar degeneration by confocal microscope. *In vitro* experiments subsequently confirmed the existence of mitochondrial damage in sepsis. Mitochonria have been proposed as a key players in the pathogenesis of sepsis. Ultra-structural aterations of mitochondria have been found in animal species (26). Changes in mitochondrial morphology, such as fragmentation and swelling, have been observed in septic patients (27). This evidence is in line with the report that increased mitochondrial respiration and ATP synthesis can reduce oxidative stress, overcome metabolic paralysis, regenerate tissues, organs and innate and adaptive immune cells, which makes sepsis better survival (28). Some drugs targeting mitochondrial are being developed. Some known effective drugs have also been proven to work partially through mitochondrial-related pathways (29). This fact indicates MiRGs could function as therapeutic targets for sepsis patients.

We screened out three MiRGs and verified them again with our specimens. So far, there have been no in-depth studies on sepsis with these three genes. *NDUFB3* (NADH dehydrogenase (ubiquinone) 1 beta subcomplex, 3) is an important subunit of mitochondrial respiratory complex I. Our data showed *NDUFB3* played an important role in the process of mitochondrial mass imbalance in the simulated sepsis model treated with LPS. However, seldom evidence could be found concerning its functions in sepsis progression. The expression changes of *NDUFB3* lead to the production of mitochondrial reactive oxygen species (mtROS) (30). The roles of mtROS in sepsis are twofold. On the one hand, mtROS can be used as bactericidal weapon during infection. However, mtROS levels are essential to induce an effective immune response within a controlled range. When mitochondrial damage occurs, overproduction of mtROS can lead to persistent inflammation, leading to pathologic outcomes such as sepsis (31).

Reports about *BCKDHB* have focused on its effect on maple syrup urine disease (MSUD). *BCKDHB* gene is one of the main catalytic subunits of branched ketoate dehydrogenase (BCKDH) in mitochondria. Together with BCKDHA, it forms a branched α-ketoate dehydrogenase E1 complex, which can decompose branched amino acids (32). Increased branched-chain amino acid concentrations were found in a variety of insulin-deficient and resistant states. The mechanism of BCKDH is not fully understood, and the decreased activity of BCKDH may be an important cause (33). Sepsis is associated with hypermetabolism. If the hypermetabolic state persists, life-threatening multisystem organ failure may occur (34). In this case, branched-chain amino acids are important energy substrates for muscles (35, 36). Insulin resistance and inflammation-related metabolic changes in the Sepsis process may be related to branched-chain amino acid metabolism involved in *BCKDHB* (34). Protein 1 of the LETM1 domain (LETMD1), also known as HCCR-1, is a mitochondrial protein. Limited studies have shown that *LETMD1* is essential for the mitochondrial structure and thermogenic function of brown fat cells (37). *LETMD1* selectively regulates reactive oxygen generation and NF-κB activation in macrophages through MyD88, thus regulating phagocytosis and inflammatory response to lipopolysaccharide (38). Inflammatory reactions of lipopolysaccharide trigger the secretion of pro-inflammatory cytokines and other biological processes through initiating signal cascades, thus becoming an extremely important link in the process of sepsis and development, and a potential therapeutic target (39, 40).

The correlation analysis of immune cell infiltration suggests the existence of immunosuppressive and depleted microenvironments in sepsis patients. In addition, after dividing sepsis patients into two groups using consensus clustering, we observed the effect of MiRGs on the immune microenvironment of patients with Sepsis. The effect of MiRGs on immune infiltration in other diseases has been reported (41–43). We have observed that DE-MiRG screened in sepsis is associated with neutrophil immune infiltration. Compared with the control group, the levels of neutrophils in patients with sepsis were significantly higher. Neutrophils control the infection by migrating to the inflamed site and exercising their lethal role against the pathogen. In sepsis, neutrophil migration and killing capacity are decreased, resulting in an insufficient response to infections and easy collateral damage to surrounding tissues due to the decline in precision (44). In addition, increased levels of neutrophils in sepsis patients often lead to a number of harmful functions. First, the accumulation of activated neutrophils blocks the capillary lumen, leading to ischemia (45). Secondly, neutrophils that migrate to vital organs can locally release pro-inflammatory and lytic factors that cause local tissue damage (46). As a double-edged sword in the progression of sepsis, the relationship between high neutrophil levels in patients with sepsis and prognosis needs to be further investigated. The consensus clustering further indicated the correlation of these three mitochondria-related hub genes with a high level of neutrophils in sepsis. With increasing evidence that neutrophils may be a promising therapeutic target for sepsis treatment (47), the possibility of MiRGs as therapeutic targets needs to be further explored.

Our data also showed high levels of eosinophils in patients with sepsis and significant differences in eosinophil expression among MiRGs subgroups of sepsis patients. This is consistent with the previously reported conclusion that there is a positive association between increased eosinophilic counts and sepsis compared with non-septic trauma patients admitted to the ICU (48). Currently, there is no consensus on the role of eosinophils in sepsis. Low levels of peripheral eosinophil activity have been reported to be associated with poor survival in sepsis (49). One explanation is that the type 2 immune response is related to eosinophilia. It can balance the pro-inflammatory response of sepsis due to type 1 immune response disorder. Thus, the lack of eosinophils may be a manifestation of an immune imbalance and may thus trigger the secretion of pro-inflammatory cytokines, leading to poorer outcomes (49). In addition, when stimulated by bacterial lipopolysaccharide, eosinophils, can release mtDNA and form extracellular structures with granular proteins that can bind to and kill bacteria, thus contributing to antibacterial defense (50). In general, studies on sepsis, MiRG and the interaction with eosinophils are superficial and need further investigation.

In summary, we identified three MiRGs (*BCKDHB*, *LETMD1*, and *NDUFB3*) by the machine learning algorithm. Subsequently, the role of these hub genes in immune cell infiltration was studied to further understand the immune mechanism in the pathogenesis of sepsis. Differences in the immune micro-environment between subgroups of patients with sepsis provide innovative insights into personalized immunotherapy for sepsis. By qPCR, the high expression of *NDUFB3* in sepsis was verified by our clinical specimens. *In vitro* experiments showed *NDUFB3* plays an important role in the process of mitochondrial mass imbalance in the LPS-simulated sepsis model. This study provides new ideas and targets for the intervention and treatment of sepsis. Although we provide risk stratification and potential intervention targets by expanding the pool of sepsis biomarkers including NDUFB3, the scope of application of a single biomarker is limited due to the complexity of sepsis etiology (51). In the future, combined with other studies, the sepsis risk stratification marker pool will be further expanded. Gene array analysis will help reduce the bias of gene selection, in order to accurately evaluate different sepsis subtypes, stages of disease progression and treatment intensity selection (52, 53). There are for sure shortcomings in this study. We have not been able to conduct further experimental verification of the molecular mechanism of *NDUFB3* caused mitochondrial mass imbalance. The immune infiltration results were a correlation rather than a more accurate causal analysis. Further in-depth explorations about the role of MiRGs could be done in the future.

## Data availability statement

The datasets presented in this study can be found in online repositories. The names of the repository/repositories and accession number(s) can be found in the article/**Supplementary Material**.

## Ethics statement

This study protocol was approved by the Ethics Committee of the Army Medical University and was registered by the Chinese Clinical Trial Registry (ChiCTR2200055772). The patients/participants provided their written informed consent to participate in this study.

## Author contributions

JZ, LZ and XC contributed the data collection and data analysis. QS and HS conceived the original ideas and composed this manuscript. LF contributed the table and figures of this manuscript. QS and HS contributed equally to this article and all authors contributed to the article and approved the submitted version.
