# Supplementary material for: Identification and experimental validation of mitochondria-related genes biomarkers associated with immune infiltration for sepsis
Source: Front Immunol. 2023 May 9;14:1184126. doi: 10.3389/fimmu.2023.1184126 (PMC10203506; doi:10.3389/fimmu.2023.1184126)
Supplement: Supplementary file 1 [file DataSheet_1.docx]

Supplementary Table 1: The inclusion and exclusion criteria of sepsis patients.

| Inclusion criteria | 1. Diagnosed as sepsis samples. 2. The patient informed consent and participated voluntarily. 3. 18-80 years old. 4. Register general medical conditions. |
| --- | --- |
| Exclusion criteria | 1. Age>80 years or ≤18 years were excluded. 2. Previous history of chronic heart disease, liver and kidney diseases. 3. Pregnant or lactating women. 4. Hyperlipidemia, diabetes, or other metabolic diseases. 5. Complicated with cardiovascular, liver, kidney and hematopoietic system and other serious primary diseases. 6. Combined with psychiatric diseases. 7. Long-term use of sedative drugs or alcohol. 8. Complicated with tumor or immune deficiency and other diseases that have a greater impact on immunity. 9. Patient or family refusal to be involved in the study. |

Supplementary Table 2. PCR primer sequences of hub genes.

| Genes | Forward primers | Reverse primers |
| --- | --- | --- |
| BCKDHB | *TATCGCTATCGCTCTGGGGA* | *CCACCTTGATTCCTGGGCAA* |
| LETMD1 | *CCCTGGACATTTTGTCACCC* | *AAGCTTTGAAGACCGAGGGG* |
| NDUFB3 | *GCTGCAAAAGGGCTAAGGGA* | *ACAGCTACCACAAATGCAGC* |
| β-actin | *GTGGATCAGCAAGCAGGAGT* | *ATCCTGAGTCAAGCGCCAAA* |

**
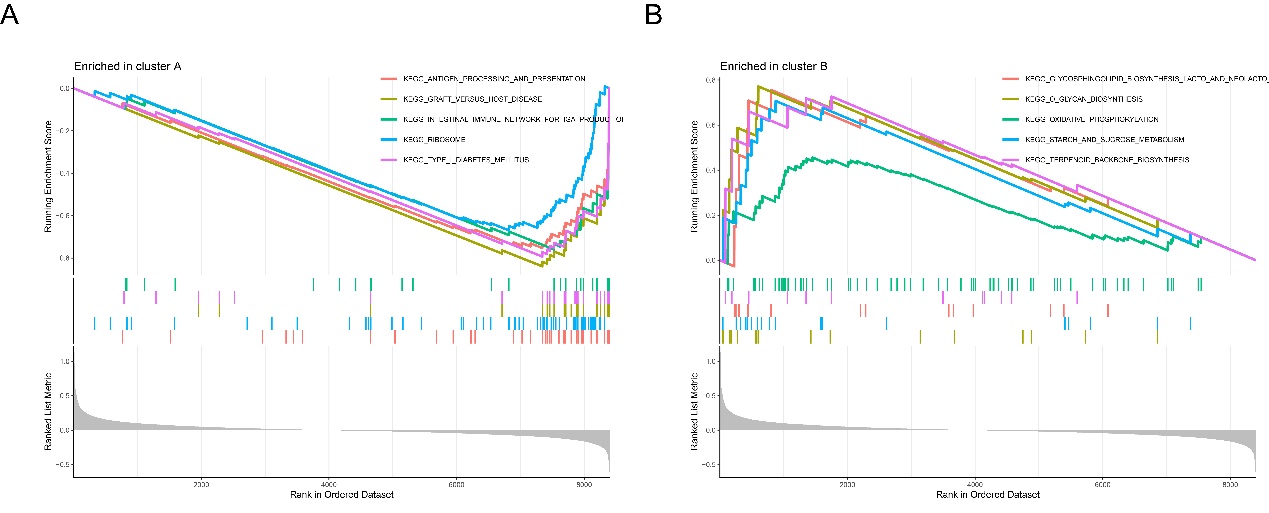
**

Supplementary Figure 1. GSEA analysis of DEGs in Cluster A and Cluster B groups.


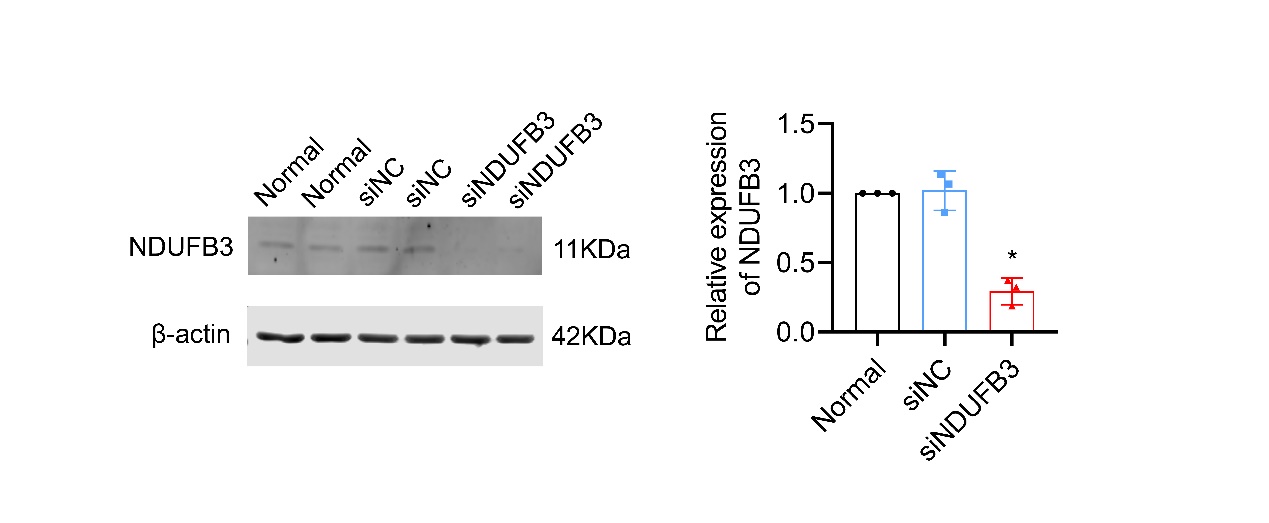


Supplementary Figure 2. Western blotting result showing the inhibitory effect of siNDUFB3 to the expression of NDUFB3 (n = 3). *P< 0.05 as compared with the Normal group.
